# Assessing Screening Guidelines for Cardiovascular Disease Risk Factors using Routinely Collected Data

**DOI:** 10.1038/s41598-017-06492-6

**Published:** 2017-07-26

**Authors:** Jaspreet Pannu, Sarah Poole, Neil Shah, Nigam H. Shah

**Affiliations:** 1 School of Medicine, Stanford University, Stanford, CA USA; 2 Stanford University Center for Bioinformatics Research, Stanford, CA USA; 3 Department of Pathology, School of Medicine, Stanford University, Stanford, CA USA

## Abstract

This study investigates if laboratory data can be used to assess whether physician-retesting patterns are in line with established guidelines, and if these guidelines identify deteriorating patients in a timely manner. A total of 7594 patients with high cholesterol were studied, along with 2764 patients with diabetes. More than 90% of borderline high cholesterol patients are retested within the 3 year recommended period, however less than 75% of pre-diabetic patients have repeated tests within the suggested 1-year time frame. Patients with borderline high cholesterol typically progress to full high cholesterol in 2–3 years, and pre-diabetic patients progress to full diabetes in 1–2 years. Data from routinely ordered laboratory tests can be used to monitor adherence to clinical guidelines. These data may also be useful in the design of adaptive testing strategies that reduce unnecessary testing, while ensuring that patient deterioration is identified in a timely manner. Established guidelines for testing of total serum cholesterol for hypercholesterolemia are appropriate and are well-adhered to, whereas guidelines for glycated hemoglobin A1c testing for type 2 diabetes mellitus could be improved to bring them in line with current practice and avoid unnecessary testing.

## Introduction

Cardiovascular disease (CVD), including ischemic heart disease and stroke, is the primary cause of death worldwide, leading to 32% of all deaths worldwide in the year 2013^1^. CVD costs the United States more than $315 billion annually, consuming almost one in every six dollars spent on healthcare^2^. Prevention of CVD would markedly decreases costs to the health system, and would improve quality of life at a population level.

Type 2 diabetes mellitus and hypercholesterolemia are well established modifiable risk factors for CVD^3, 4^, and are easily diagnosable with simple laboratory tests^5^. To identify at-risk patients in the United States, professional organizations publish screening guidelines outlining the utilization of laboratory testing. Well-established guidelines are in place for screening of both hypercholesterolemia and diabetes (Table 1).

**Table 1:** Guidelines for performing screening and monitoring tests^18, 19^. Additional patient risk factors include hypertension, obesity, smoking, and family history of premature disease in a first-degree relative.

Laboratory test name	Previous result	Additional risk factors	Repeat test indicated
Total serum cholesterol	Normal (<200 mg/dL)	Absent	Begin screening at age 20, repeat every 4 to 6 years
		Present	Within 3 years
	Borderline high cholesterol (200–239 mg/dL)		Within 3 years
	High cholesterol (≥240 mg/dL)		Within 3 years
Glycated hemoglobin A1C	Normal (<5.7%)	Absent	Begin screening at age 45, repeat every 3 years
		Present	Begin screening at age 18, repeat every 3 years
	Prediabetic (5.7–6.4%)		Within 12 months
	Diabetic (≥6.5%)		Within 4 months

Despite the presence of these guidelines, approximately 6.2% of US adults have undiagnosed hypercholesterolemia^6, 7^. Similarly, it is estimated that another 6.3 million adults have undiagnosed diabetes^8^. Screening guidelines are designed for early detection of a disease, which has been shown to reduced morbidity and mortality in both diabetes mellitus and hypercholesterolemia patients^9–15^. The large number of patients with undiagnosed hypercholesterolemia and diabetes mellitus make it necessary to study both guideline adherence as well as their ability to identify patients before they deteriorate.

In this paper, we use routinely collected laboratory data to assess whether established guidelines for screening of hypercholesterolemia and type 2 diabetes mellitus capture deteriorating patients in a timely fashion that allows early lifestyle intervention for possible prevention of CVD. Although many laboratory parameters are collected on a routine basis, we have selected the two tests most commonly used at this institution to identify cardiovascular disease risk for a proof-of-concept study. We hope to show that access to large amounts of data allows differences between recommended retesting and actual practice patterns to be identified^16^, irrespective of the disease being screened for. Based on these findings, changes can be made to adjust retesting time, or to encourage physician adherence to guidelines in order to minimize the differences between recommended and actual retesting.

## Methods

The Stanford Translational Research Integrated Database Environment (STRIDE) was the primary source of data used for this project^17^. STRIDE contains data from 2 million pediatric and adult patients over 18 years, and includes 25 million clinical encounters, 48 million ICD9-coded inpatient and outpatient diagnoses, and 157 million laboratory test numeric results. Experimental protocols for this project were approved by the Stanford Institutional Review Board (IRB). All methods were performed in accordance with guidelines and regulations for research involving patient data, as provided by the Stanford University IRB and the Center for Bioinformatics Research. Informed consent was not required for this study. All data from STRIDE is compliant with the Health Insurance Portability and Accountability Act (HIPAA); all information that could lead to identification of a study participant has been removed by a third party before it is accessed by any researcher involved in this study.

We extracted laboratory data related for two tests of interest, namely total serum cholesterol, and glycated hemoglobin A1c. These tests defined two cohorts of patients, a high glycated hemoglobin A1c cohort, at risk of developing type 2 diabetes mellitus, and a high total serum cholesterol cohort, at risk of developing hypercholesterolemia. First, all patients with at least one result for the test of interest were selected. This study examines the time taken for a patient to progress from a pre-disease state, such as borderline hypercholesterolemia or pre-diabetes, to a full disease state; therefore inclusion in the cohort required at least one test result in the pre-disease range before a test result in the full-disease range. The pre-disease and full-disease test result ranges are defined using published guidelines^18, 19^, and are shown in Table 1. The characteristics of each cohort are shown in Table 2.

**Table 2:** Demographics of borderline high cholesterol and pre-diabetic cohorts. Age is at first pre-disease lab result.

	Borderline High Cholesterol Group (n = 7594)	Pre-Diabetes Group (n = 2764)
Age, years		
Mean	52	59
Range	11–88	17–88
Sex		
Male	3291	1548
Female	4303	1216
Race		
Asian	1086	552
Black	202	159
Native American	30	9
Pacific Islander	47	39
White	4315	1282
Other	739	394
Unknown	1175	329

The first investigation determined the time elapsed between the first recorded pre-disease test result and the next follow up test. This “time to retest” was compared with the recommended retest interval for patients in the pre-disease range. Patients were stratified into three groups based on the value of their first pre-disease test result.

The second investigation examined the time between the first pre-disease result and the transition to a full disease state, with patients stratified by test result value as before.

### IRB approval

This study was approved by Stanford University’s Institutional Review Board prior to data collection.

### Data Availability

All data and associated protocols used in this study are available to readers. Please contact the corresponding author.

## Results

**Figure 1:**
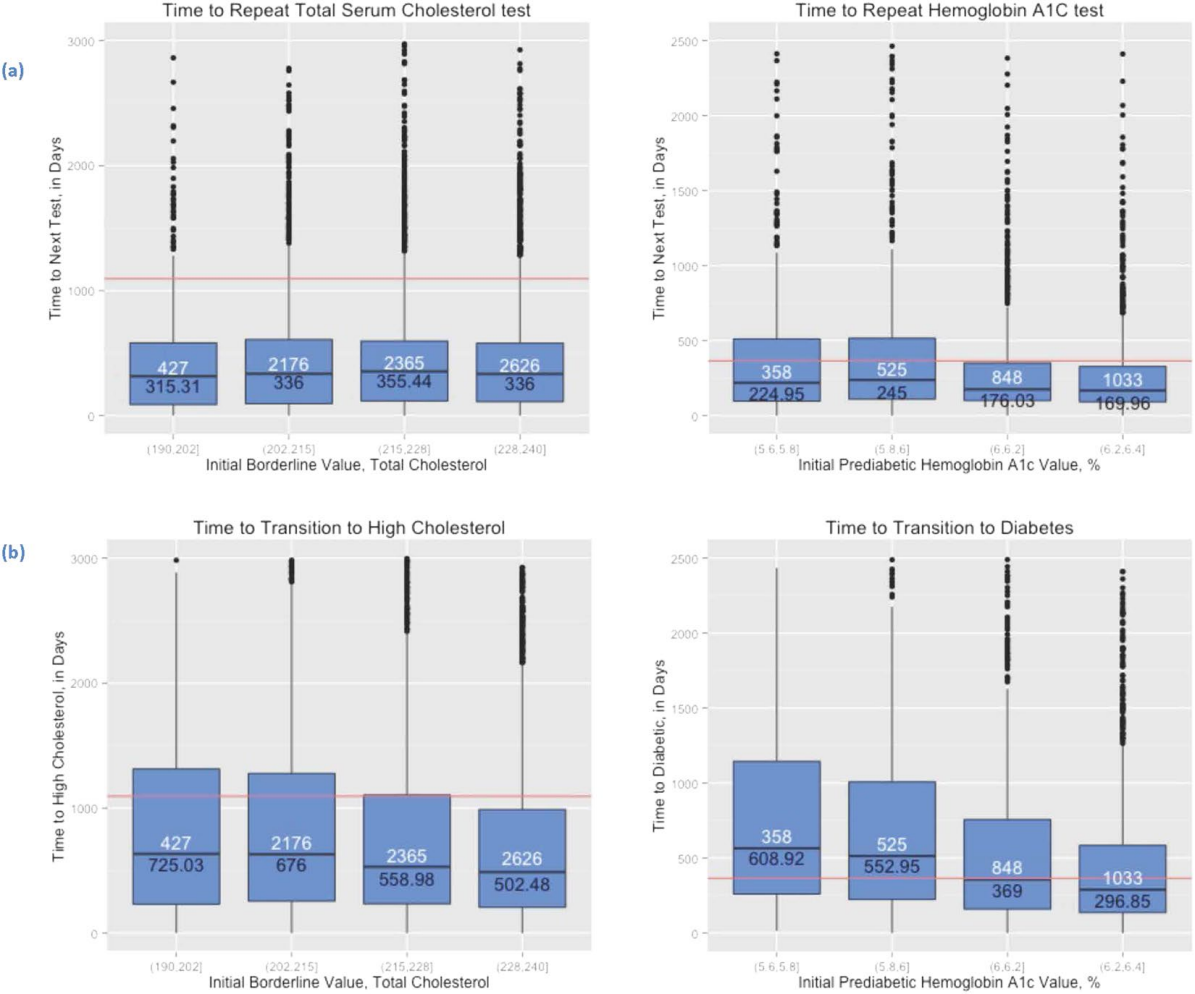
(**a**) Time between first pre-disease test result and retest. Red line indicates the recommended time to retest for pre-disease patients. Median time to retest is shown in black, with the number of patients shown in white. 91% of patients (n = 6892) are tested for total cholesterol within guidelines. 73% of patients (n = 2009) are tested for HA1c within guidelines. (**b**) Time between first pre-disease test result and transition to full disease state. Red line indicates the recommended time to retest for pre-disease patients. Median time to retest is shown in black, with the number of patients shown in white.

Figure 1(a) shows the time elapsed between the first recorded pre-disease test result and the first follow-up test, for both total serum cholesterol and glycated hemoglobin A1c tests. Patients with newly detected borderline-hypercholesterolemia are routinely retested within the recommended 3 years, with 91% of retests occurring during this period. However, only 73% all repeat hemoglobin A1c tests for patients with newly detected pre-diabetes occur within the recommended 12 months.

Figure 1(b) shows the time between the first recorded pre-disease test result and the transition to a full disease state. Note that transition can only be detected if a retest is actually performed. 71.8% of patients with borderline cholesterol transition to hypercholesterolemia in 3 years, whereas 48% of the patients classified as pre-diabetic transition to type 2 diabetes in 12 months. Thus, borderline high cholesterol patients are highly likely to transition to hypercholesterolemia within 2–3 years. Whereas a large fraction of patients with glycated hemoglobin A1c levels classifying them as pre-diabetic transition to full type 2 diabetes mellitus after 12 months. In both disease groups, we see a pattern of faster time to transition in patients with higher initial test results.

## Discussion

The pattern of repeat testing of total serum cholesterol levels shows that the guidelines in place for this test are being well adhered to in practice. Repeat tests are almost always within the recommended 3 years for borderline hypercholesterolemia patients, with over 91% of patients being retested within this period. The guidelines that are in place are fulfilling their purpose, ensuring that the 71.8% of patients who transition to a full disease state within 3 years are identified in a timely manner.

The guideline for repeat testing of glycated hemoglobin A1c levels is not well followed in practice, with only 73% of the patient cohort having a repeat test within the recommended 12 months. However, only 48% of the cohort transitions from pre-diabetes to full type 2 diabetes mellitus within this period.

If we consider the pattern seen in the repeat testing of total serum cholesterol levels to be ideal (i.e. the majority patients are tested at an interval that will identify the majority of those that transition), the recommended time to retesting of glycated hemoglobin A1c should be extended to 1000 days. This would reduce the number of recommended tests during this period from three to one, reducing costs and inconvenience to patients, while still identifying most patients who progress to full type 2 diabetes mellitus in a timely manner.

An adaptive testing strategy could be implemented, where re-test time is dependent on the first test result. Only 63.3% of patients with an initial pre-disease glycated hemoglobin A1c value between 5.7% and 6% currently receive a repeat test within the recommended 12 months, and only 33.7% transition to full type 2 diabetes mellitus within this period. This result may be reflection of the “wisdom of the crowd”, in that physicians already know the low probability of transitioning to full disease state after an initial low result^20^. Extending the recommended time to retest for patients with glycated hemoglobin A1c values in this range would bring the guidelines in line with practice, without impacting the ability to identify patient deterioration.

## Conclusion

Practice-based evidence shows that established guidelines for screening of hypercholesterolemia are well-suited, but that guidelines for type 2 diabetes mellitus could be improved to remove unnecessary testing. An adaptive testing strategy would bring guidelines in line with actual practice, removing the recommendation for annual testing without impacting identification of patient transitions. Ultimately, the methods outlined in this proof-of-concept study may be extended to other routinely performed screening laboratory tests. Thus, laboratory data represents a valuable opportunity to identify the efficacy of screening guidelines and devise adaptive testing strategies.
